# Promoting dairy consumption behavior in the school setting: an experiment based on the transtheoretical model

**DOI:** 10.1186/s40795-023-00736-7

**Published:** 2023-06-28

**Authors:** Nooshin Rouhani-Tonekaboni, Sara Sabrkonandeh, Asieh Ashouri, Parisa Kasmaei, Mehdi Mirzaei-Alavijeh

**Affiliations:** 1 Department of Health Education and Promotion, Research Center of Health and Environment, School of Health, Guilan University of Medical Sciences, Rasht, Iran; 2 School of Health, Guilan University of Medical Sciences, Rasht, Iran; 3 Cardiovascular Diseases Research Center, Department of Cardiology, Heshmat Hospital, School of Medicine, Guilan University of Medical Sciences, Rasht, Iran; 4 Social Development and Health Promotion Research Center, Health Institute, Kermanshah University of Medical Sciences, Kermanshah, Iran

**Keywords:** Dairy products, Students, Nutritional Education, The Transtheoretical Model

## Abstract

**Background:**

Nutrition education is a key component of health promotion programs which leads to the improvement of students’ nutritional behaviors. The transtheoretical model (TTM) is one of the models extensively used in changing people’s behaviors. This study aimed to change the dairy consumption behavior based on the TTM in female students.

**Methods:**

A controlled trial was conducted with 159 female students (intervention group: 56; control group: 103) in the 10-11th grades from two public schools in Soumesara city located in the west of Gilan Province, Iran. Demographic characteristics, knowledge, TTM constructs and stage of change of dairy consumption were collected using a valid and reliable researcher-made questionnaire. Data were gathered before and one month after the educational intervention. Chi-square test, t-test and ANCOVA were used to analyze the data and a p-value < 0.05 was considered statistically significant.

**Results:**

Fifty-two students from the intervention and 93 from the control group completed the study. Only 15% of the students were in the action or maintenance stages of dairy consumption. After the intervention, mean scores of behavioral processes of change, cognitive processes of change, decisional balance, and self-efficacy improved in the intervention group (P < 0.05 for all). Also, 37% and 16% of the participants in the intervention and control groups, respectively, were in the action or maintenance phase (P < 0.001).

**Conclusions:**

This study showed that implementing an intervention based on the TTM would positively affect students’ dairy consumption behaviors. Also, it is suggested that the TTM be assessed in terms of other daily nutritional needs in students to promote their nutritional behaviors.

**Trial Registration:**

The study was registered in the Iranian Registry of Clinical Trials (IRCT) (available online at https://en.irct.ir/trial/50003) on April 11, 2020 with the number IRCT20200718048132N1 and was approved by research ethics committee of Guilan University of Medical Sciences, Iran.

**Supplementary Information:**

The online version contains supplementary material available at 10.1186/s40795-023-00736-7.

## Background

Recent studies have shown that dairy consumption is useful for building muscles, lowering blood pressure, reducing blood lipids and preventing tooth decay, diabetes, cancer and obesity [1].

Dairy products have important micronutrients such as calcium, potassium, vitamin D and protein which play an important role in boosting the immune system, maintaining normal functioning of the body and ensuring good health as a result. For example, calcium consumption has a positive effect on bone health and is essential for bone development at an early age [2, 3]. Also, calcium is the most abundant mineral in the body; in fact, approximately 99% of calcium is found in bones and teeth [4]. According to age, the required amount of this element in the human body varies from 1000 to 1500 mg [5], and it is interesting to note that 1300 mg is recommended for ages 9–18 [4]. Given that approximately 11 million of Iran’s population are adolescents based on the 2016 population census and that the increasing population of this age group mostly belongs to females, it is important to pay much more attention to girls’ health programs [6, 7].

In fact, female nutritional status is more important than male nutritional status because girls are future mothers; therefore, their understanding of nutritional concepts affects not only their own health but also their children and family’s health in the future [8]. In this regard, adequate nutritional calcium is needed both prior to and during their puberty to achieve optimal bone mass and density; calcium intake in girls causes bone density and protects them against osteoporosis in the postmenopausal years [4]. In the United States, a large percentage of people still consume less than adequate amount of minerals such as calcium and iron [4]. In Iran, similar to many developing countries, poor nutrition among students is increasing now. For example, in one study, the average level of fast-food consumption in students was reported high [9]. In another study carried out in the west of Gilan Province, it was found that only 41% of students would consume dairy products twice or more times a day [10].

However, implementing health promotion programs effectively and efficiently requires some theory-based interventions [11]. One of the most popular models in the area of behavior change is known as the transtheoretical model. This model focuses on explaining behavior change and proposes that individuals change their behavior through various stages. In the late 1970s, James Prochaska from the University of Rhode Island laid the foundation for the TTM after reviewing the various theories of psychotherapy [12]. The TTM consists of several constructs. The first construct of the TTM posits that individuals are not at the same level of preparation, and these stages indicate motivational states. These stages include pre-contemplation, contemplation, preparation, action, and maintenance. In the first stage (pre-contemplation), individuals have no intention of changing behavior in the next 6 months. During the second stage (contemplation), individuals state that they intend to take action within the next 6 months. In the third stage (preparation), one intends to perform the behavior in the near future (usually within the next 30 days). The fourth stage is the action stage in which individuals openly change their behavior and intend to keep moving forward with that behavior change for up to six months. In the fifth stage (maintenance), individuals sustain their behavior change for more than six months and attempt to prevent relapse to earlier stages [13].

The second construct of TTM includes 10 processes of change. These processes refer to activities that help the individual progress in the change stages. This construct is composed of 5 experiential and 5 behavioral processes [12]. Five experiential change processes are as follows: consciousness raising, dramatic relief, environmental re-evaluation, self-re-evaluation, and social liberation. These processes are briefly described in the following: Consciousness raising requires an increase in consciousness of the causes, consequences and treatment of a particular problem. Dramatic relief is a process that increases emotional arousal about one’s behavior and the calmness that can result from change. Environmental re-evaluation requires both emotional and cognitive components of how behavior affects a person’s living environment and how behavior change affects their living environment. Self-re-evaluation requires both emotional and cognitive components and involves assessing one’s self-concept with new behavior. Finally, social liberation refers to the opportunities and alternatives that exist to show society is supportive of the healthy behavior.

Five behavioral change processes are as follows [12]: self-liberation, counter conditioning, reinforcement management, stimulus control, helping relationships. These processes are briefly described in the following: Self-liberation includes the belief that a person is capable of change and includes commitment and recommitment to act on that change. Counter conditioning requires learning new and healthier behaviors instead of old and unhealthy ones. Reinforcement management is, in fact, a process that uses reinforcements and punishments to take steps in a specific direction. Stimulus control involves modifying the environment to increase guidelines for health behavior and reducing guidelines for unhealthy behavior. Finally, helping relationships requires the development of caring, reliable and acceptable relationships to observe health behaviors.

The third construct of the TTM is known as decisional balance. This construct involves balancing the perceived benefits and advantages (pros) of adopting a new behavior with the disadvantages or barriers (cons) of adopting it. The fourth construct of the TTM is self-efficacy. This construct refers to the individual’s self-confidence to create and maintain a change in behavior and avoid relapse [12].

The TTM has been applied in many settings, including primary care, schools, and communities [13]. Some examples in which the TTM has been used for primary prevention are increasing fruit and vegetable consumption, reducing overweight and obesity in primary care, and promoting physical activities [12]. So far, several studies have been conducted to teach healthy nutritional behaviors using the TTM in different populations. For example, Kohpeima-Jahromi et al. reported that the educational intervention based on the TTM would be successful in achieving the goal of modifying osteoporosisrelated behaviors in female students. They also found that, after the intervention, there was a significant difference in the selfefficacy and some sub-construct of the processes of change [14]. In one study of a systematic review, it was concluded that the TTM would seem to be a successful strategy for improving dietary behaviors in adolescents [15].

According to some evidence in the literature [12–15], the application of the TTM in promoting nutritional behavior in school has been successful. Given that an increase in students’ tenancy to eat unhealthy diet leads to health problems and that dairy consumption is insufficient in Iran (particularly in the west of Gilan) [10, 16], the present study aimed to change the behavior of dairy consumption in female students through the TTM.

## Methods

### Study Design, participants and recruitment

This study was a pretest-posttest quasi-experimental study conducted from September to November 2020 in public schools in Soomehsara, a city located in the west of Gilan Province, Iran. The total participants of this study were 145 female students in 10th and 11th grades studying at two public schools Soomehsara,. In a non-random allocation manner, one of the schools was assigned to be the intervention group and the other was considered the control group. This decision was made due to an easy permission granted from the management of the intervention school. The two inclusion criteria were voluntary participation and no illness, while the exclusion criteria included incomplete survey completion, absence from at least one of the educational sessions, changing school or immigration, and having a special diet of dairy consumption prescribed by an expert.

**Fig. 1:**
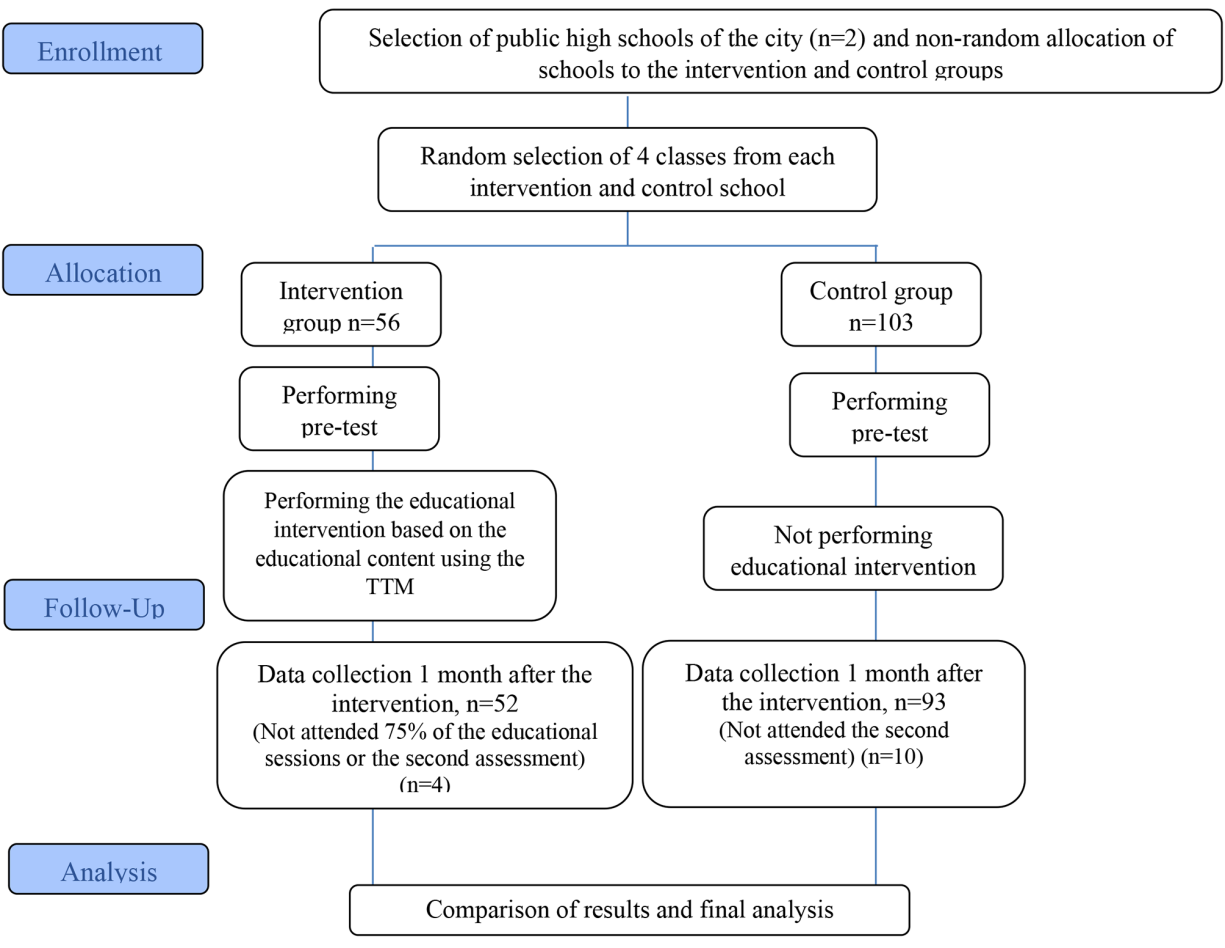
Flowchart of the study design and participants

Based on the Ghaffari et al.‘s study [17], the sample size was calculated for the effect size of 0.6. Considering alpha level of 0.05 and power of 0.8, 49 students in each group were required. Four classes (N = 56) were randomly selected from the intervention school and 4 classes (N = 103) were randomly chosen from the control school. Figure 1 depicts the flowchart of the study design process. All students in the class entered into the study and no selection was made. It should be noted that this study was registered in the Iranian Registry of Clinical Trials (IRCT) (available online at https://en.irct.ir/trial/50003) on April 11, 2020 with the number IRCT20200718048132N1 and was approved by research ethics committee of Guilan University of Medical Sciences, Iran. Also, this study adheres to CONSORT guidelines (http://www.consort-statement.org/).

### Instruments

Data collection tool (Supplementary file 1) was a questionnaire consisting of demographic characteristics (including students’ education level, place of residence, number of family members, birth rank, parents’ occupation, and education level), items of the constructs of the TTM (explained in Table 1), and students’ knowledge.

The cognitive processes of change construct included five subscales of consciousness raising, dramatic relief, self-re-evaluation, environmental re-evaluation, and social liberation, each included two items. In addition, the behavioral processes included five subscales of self-liberation, counter conditioning, reinforcement management, stimulus control, and helping relationships, each included two items.

The decisional balance score was calculated by the subtraction of the dairy consumption cons from the dairy consumption pros sum scores. Higher scores then would indicate that the students preferred the pros of dairy over its cons.

**Table 1:** Constructs, sources, validity and reliability of items used in the study

Construct	Subscale and number of items	Source	Validity		Reliability	
			I-CVI	S-CVI	ICC	Cronbach’s alpha
Stages of change	One item with five options of “I currently consume less than 2 units* of dairy per day, and I do not intend to increase its consumption within the next 6 months” (pre-contemplation phase); “I currently consume less than 2 units* of dairy per day, but I plan to increase it within the next 6 months” (contemplation phase); “I currently consume less than 2 units* of dairy per day, but I plan to increase it to 2 to 3 units* per day within the next 30 days” (preparation phase); “I have been consuming 2 to 3 units* of dairy products daily for the past 6 months” (action phase); “I have been consuming 2 to 3 dairy products a day for more than 6 months” (maintenance phase)	Gulliver and Horwath [18]	1.00	1.00	1.00*	---
Processes of change	including 10 items of cognitive processes of change and 10 items of behavioral processes of change evaluated with Likert-type options from never (1 score) to ever (5 scores)	made by the authors	0.72	0.93	0.97	0.88
Decisional balance	Including 10 items related to the pros and 12 items related to the cons of dairy consumption subscale with options of “It is not important at all” (1 score); “it is a little important” (2 scores); “it is somewhat important” (3 scores); “it is important” (4 scores); and “it is very important” (5 scores)	Gulliver and Horwath [19] and Vahedi et al. [20]; with adapt of some items	0.75	0.94	0.98	0.88
Self-efficacy	10 items with options of “I’m not sure at all” (1 score); “I’m somewhat sure” (2 scores); “I’m sure” (3 scores); “and I’m totally sure” (4 scores)	Horan et al. [21] and the Gulliver and Horwath [19]; with adapt of some items	0.89	0.99	0.98	0.88

I-CVI indicates item content validity index; S-CVI, scale content validity index; ICC, intra-class correlation.

Each unit of dairy products means a glass of milk, a bowl of yogurt, or 45 to 60 g of cheese.

* Coefficient of kappa agreement was reported.

As most of the items were extracted from papers translated into Persian through forward-backward translation method, the validity of the questionnaire was confirmed by a panel of experts, consisting of 7 specialists in health education and promotion, one specialist in nutrition, and one specialist in epidemiology. The item and scale content validity index (I-CVI and S-CVI) were calculated as described in the literature [22]. These indexes rate the agreement between the panel of experts about the clarity and relevancy of the items and scale. There result of the analysis confirmed the content validity of the scale.

To assess the reliability of the items, the questionnaire was completed by 20 students (similar but outside the study participants). Cronbach’s alpha coefficient, Kappa agreement coefficient and intra-class correlation coefficients (ICC) of test-retest method for a 2-week interval were calculated, which confirmed the reliability of the questionnaire (Table 1).

The items of knowledge in the questionnaire were adopted from the standardized knowledge questionnaire [17]. The values of I-CVI, S-CVI, intra-class correlation, and Cronbach^’^s alpha coefficient were 0.74, 0.94, 0.99 and 0.93, respectively. Knowledge was assessed with 12 items with the score range of 0–24. The range of responses to the items included incorrect (0 score), I do not know (1 score) and correct (2 score). Scores of 0–12 for weak knowledge, 12–18 for moderate knowledge and 18–24 for desirable knowledge were considered. Higher scores would then indicate higher knowledge.

Prior to this study, the participants completed a checklist about informed consent. Then, the students of both groups completed the questionnaires. Then, the students in the intervention group were provided with an educational intervention inspired by the TTM. One month after the educational intervention, the questionnaires were completed by the students in the intervention and control groups.

### Educational intervention

The intervention group was divided into 6 subgroups with 8–9 students. The intervention program was conducted in 4 sessions (each session: 45 min.) for each subgroup. The educational sessions were held for each group of students one day a week considering all the health protocols for the students (i.e. wearing a mask, considering social distance and having sanitizers).

In the intervention program, all the constructs were used directly through the lecture method, and the participatory methods such as question and answer, group discussion, and brainstorming. This program was conducted based on the constructs with low scores assessed in all participants at the beginning of the study. To increase the effectiveness of the program, some slides, pamphlets and posters were also used.

The educational content of each session included general and specific trainings concerning the students’ stages of change in dairy consumption. The general training strategies we used in the program are explained below. We drew the students’ attention to the program by raising their awareness on the importance of nutrition during adolescence. We explained about food pyramid and the role of each food group in health. In addition, we informed them about the recommended intake of milk and its products, the side effects of insufficient calcium intake and also the diseases associated with low dairy consumption. We finally provided some tutorials with easy-to-understand explanations and instructions to follow. The specific training was performed to improve the TTM constructs regarding the expression of some issue which are listed in the following:

1. The importance of dairy consumption in health.
2. The role of dairy consumption in reducing non-communicable diseases.
3. The role of calcium in the body.
4. The role of dairy products in the prevention of osteoporosis.
5. The importance of dairy consumption during adolescence.
6. Recognizing individual and family barriers to dairy consumption and finding solutions to reduce the perceived barriers to dairy consumption.
7. Finding ways to improve perceived self-efficacy to increase daily dairy consumption by interviewing successful people.
8. Identifying and emphasizing individual ability and modeling on friends and relatives.
9. Enhancing change processes by increasing environmental re-evaluation, self-re-evaluation, social liberation, self-liberation, counterconditioning, reinforcement management, stimulus control, helping relationships (Table 2).

Having excluded the incomplete questionnaires and the students absent on the intervention session, the data analyses were performed on 145 students (52 in the intervention group and 93 in the control group).

**Table 2:** The objectives and educational content of the intervention sessions

	General goals	Specific goals (At the end of the curriculum, students can do the following:)	Teaching methods	Educational media
First session	1. Increasing students’ consciousness about the study	- Explain the purpose of the present study briefly. - Participate actively during the study and receiving educational intervention.	1. Lecturing with the use of slides 2. Question and answer 3. Group discussion	Video projector, slides, whiteboard, and educational pamphlets
	2. Raising students’ consciousness about the importance of dairy consumption	- Define the dairy group. - Express the recommended daily amount (unit) of dairy products. - Name the best food sources for the body’s calcium supply. - Explain the role of dairy products in body health. - Explain the role of dairy products in preventing osteoporosis.		
Second session	1. Raising the consciousness level of students’ perceived benefits of consuming dairy products	- Explain the health benefits of dairy products. - Name the positive effects of dairy consumption on bone growth and strength. - Name the positive effects of dairy consumption on the growth and strength of teeth. - State at least 4 benefits of milk and dairy consumption.	1. Lecturing with the use of slides 2. Question and answer 3. Group discussion 4. Brainstorming	Video projector, Whiteboard, Slides
	2. Recognizing individual and family barriers to dairy consumption	- Explain the perceived barriers to dairy consumption. - Separate perceived individual, environmental, and family barriers.		
	3. Finding solutions to reduce perceived barriers to dairy consumption	- Explain the methods of overcoming individual barriers to dairy consumption. - Describe the ways of overcoming family barriers to dairy consumption.		
Third session	1. Raising students’ consciousness to increase their daily consumption of dairy products	- Express the importance of dairy consumption - Express the positive results of dairy consumption.	1. Lecturing with the use of slides 2. Role playing 3. Group discussion	Video projector, Whiteboard, Slides
	2. Increase students’ emotional arousal about dairy consumption	Use methods that provoke emotional arousal in dairy consumption.		
	3. Recognizing the impact of students’ dairy consumption behavior on their living environment	Explain the impact of dairy consumption on their living environment.		
	4. Increasing students’ assumption of dairy consumption	Explain their assumption of dairy consumption.		
	5. Increasing students’ belief in their ability to consume dairy products and their commitment	Explain ways to increase commitment to dairy consumption.		
	6. Informing students to consume dairy products and replace it with undesirable nutritional behaviors	Explain ways to replace dairy consumption with undesirable nutritional behaviors.		
	7. Developing reinforcement and punishment in dairy consumption	- Explain the methods of strengthening dairy consumption. - Explain the punitive methods in consuming dairy products.		
	8. Providing and creating guidelines for dairy consumption in students	Explain ways to create guidelines for dairy consumption.		
	9. Using social opportunities in dairy consumption	Take advantage of social opportunities in dairy consumption.		
	10. Increasing students’ knowledge of social norms that support dairy consumption	- Know the sources of support to increase dairy consumption. - Use the support of family and teachers to increase dairy consumption.		
Fourth Session	Increasing students’ perceived self-efficacy to increase daily dairy consumption	- Explain ways to improve perceived self-efficacy to increase dairy consumption. - Reduce unpleasant feelings about dairy consumption and use verbal persuasion. - Increase their daily dairy consumption. - Use milk and dairy products for breakfast. - Use milk and dairy products for snacks. - Demonstrate this behavior by observing the behavior of successful people. - Use practical guides such as pamphlets and booklets to increase self-efficacy for dairy consumption. - Use booster messages to increase self-efficacy for dairy consumption.	1. Lecturing with the use of slides 2. Question and answer 3. Interviewing with a believable role model	Posters, educational pamphlets, slides

### Data Analysis

Data were analyzed using SPSS software version 23.0 (IBM Corp., Armonk, NY). Mean (standard deviation) and median (range) were used to describe the quantitative data, and frequency (percentage) was used to describe the qualitative data. Normal distribution of the quantitative variables was assessed using Q-Q plot. Chi-square test independent two-sample t-test or Mann-Whitney U test was performed to examine the comparability of the intervention and pre-intervention control groups in terms of demographic variables and constructs at baseline assessment. Paired t-test or Wilcoxon signed-rank tests were used to evaluate the changes before and after the intervention for each of the two groups.

As for the evaluation of the effect of the educational intervention, two independent sample t-tests and Mann-Whitney U tests were used to compare the data in terms of the constructs of the transtheoretical model and the stages of behavior change in the two groups before and after the intervention. The adjusted means of each construct between the two groups were compared using analysis of covariance (ANCOVA), so that the measured values before the intervention were adjusted. A significance level of 0.05 was considered for all analyses.

## Results

In total, 145 high-school female students participated in this study (Table 3). Eighty-four (58%) students were in the 11th grade and sixty-one (42%) were in the 10th grade. Most of the students (71%) were in families with 4 or more members and about half of the students (53%) were first-born children. 70% of the students lived in the city. Almost all (94%) students’ mothers were housewives and their education level (47%) was high school diploma or higher. The education level of 53% of the fathers was high school diploma or higher and approximately 53% of the fathers were self-employed (Table 3).

At the beginning of the study, except for parents’ education (P < 0.001 and P = 0.011) and living location (P = 0.028), none of the other demographic characteristics differed between the two groups significantly (P > 0.05 for all, see Table 3). In the intervention group, more students lived in the city and their parents’ education level was higher than those in the control group (Table 3).

**Table 3:** Demographic characteristics of the students

Characteristic	Level	Total (n = 145)	Intervention (n = 52)	Control (n = 93)	P-value
Grade	Tenth	61(42)	18(35)	43(46)	0.174
	Eleventh	84(58)	34(65)	50(54)	
the number of family members	Tow	6(5)	4(8)	2(2)	0.233
	Three	36(24)	11(21)	25(27)	
	Four and more	103(71)	37(71)	66(71)	
Location	City	102(68)	31(60)	71(76)	0.028
	Rural	43(32)	21(40)	22(24)	
Birth rank	One	77(53)	30(58)	47(51)	0.120
	Two	40(28)	17(32)	23(25)	
	Three	22(15)	3(6)	19(20)	
	Four and more	6(4)	2(4)	4(4)	
Father’s job	Manual worker	19(15)	10(20)	9(10)	0.084
	Employee	23(14)	3(6)	20(22)	
	Self-employed	73 (52.5)	28)56(	45(49)	
	Farmer	15(11)	6(12)	9(10)	
	Other	11(7.5)	3(6)	8(9)	
Mother’s job	Housewife	136(93)	)47 (90)	89(96)	0.400
	Employee	2(1.5)	1(2)	1(1)	
	Self-employed	3(1.5)	1)2)	2(2)	
	Other	4(4)	3(6)	1(1)	
Father’s education	Primary or less	29 (21)	12(24)	17(18)	0.050
	Secondary	90(65)	35(70)	55(60)	
	Tertiary	23(14)	3(6)	20(22)	
Mother’s education	Primary or less	29(20)	16(31)	13(14)	0.029
	Secondary	104(72)	34(65)	70(76)	
	Tertiary	12(8)	2(4)	10(10)	

Data are reported in the form of frequency (percentage).

Differences from the total number was due to the missing data.

Percentage is reported in total or in total number of subgroups.

The description of dairy consumption stages of change was described in both groups as shown in Table 4. At the beginning of the study, about 15% of the students were in the action or maintenance stages and there was no difference between the groups (P = 0.261). One month after the educational intervention, the results showed that education had been effective in changing the stages of dairy consumption. One month after the educational intervention, 7 (13%) students of the intervention group were at the pre-contemplation stage, 7 (13%) at the contemplation stage, 19 (37%) at the preparation stage, 13 (25%) at the action stage and 6 (12%) at the maintenance stage. In other words, about 37% of the students of the intervention group were in the action or maintenance stages. In the control group, only 16% of the students were in the action or maintenance stages (Table 4), and this showed that there was a significant difference between the two groups (P < 0.001).

**Table 4:** Description of the students’ dairy consumption stages of change for each group before and after the intervention

	Control group (n = 93)			Intervention group (n = 52)	
	Before the intervention	One month after the intervention		Before the intervention	One month after the intervention
Precontemplation	24 (26)	20 (21)		17 (33)	7 (13)
Contemplation	32 (34)	36 (39)		19 (36)	7 (13)
Preparation	23 (25)	22 (24)		9 (17)	19 (37)
Action	3 (3)	5 (5)		4 (8)	13 (25)
Maintenance	11 (12)	10 (11)		3 (6)	6 (12)

Data are reported in the form of frequency (percentage).

There is no difference between control and intervention groups in terms of dairy consumption change stages distribution before the intervention (P = 0.261, Mann-Whitney U test); however, two groups differ significantly one month after the intervention in which dairy consumption stages of change in the intervention group is higher compared to the control group (P < 0.001, Mann-Whitney U test).

The description of the TTM constructs is shown in Table 5. Scores of change process constructs, decision balance and self-efficacy were at the average level. Before the educational intervention, there was no difference between the two groups in terms of the constructs of decisional balance (P = 0.749), processes of change (P = 0.470) and self-efficacy (P = 0.893). Only the subscales of consciousness raising (P = 0.005) and the dramatic relief of cognitive change processes (P = 0.022) were different between the two groups before the intervention. Similarly, the students’ awareness score was moderate. The mean and standard deviation of the awareness score was 17.79 (2.19) and 17.59 (2.36) in the intervention and control group, respectively. In fact, there was no difference in the awareness of dairy consumption between the two groups (P = 0.525).

**Table 5:** The description of the TTM constructs measured at baseline and one month after the educational intervention

constructs	Intervention group (n = 52), mean (SD)				Control group (n = 93), mean (SD)			P**
	baseline	one month after intervention	Adj. mean (95% CI)*		baseline	one month after intervention	Adj. mean (95% CI)*	
Processes of change	52.8 (11.6)	59.4 (11.2)	60.5 (59.6–61.4)		54.45(14.5)	54.95(14.5)	54.3(53.6–55)	< 0.001
Cognitive Processes of change	28.90(7.2)	34.4(6.7)	35.4(34.8–36.1)		30.6(7.9)	30.9(7.8)	30.4(29.9–30.9)	< 0.001
Consciousness raising	4.1(1.4)	5.9(1.2)	6.3(6-6.6)		4.9(1.8)	5.3(1.7)	5.1(4.9–5.3)	< 0.001
Dramatic relief	5.8(2.3)	6.9(1.7)	7.3(7-7.6)		6.7(2.2)	6.6(2.1)	6.3(6.1–6.6)	< 0.001
Environmental re-evaluation	5.9(2.1)	7.5 (1.6)	7.6(7.3–7.9)		6.1(2.2)	6.2(2.2)	6.1(5.9–6.4)	< 0.001
Self-re-evaluation	6.4(2.4)	6.2(2.4)	7.1(6.9–7.4)		7.2(1.9)	6.3(2.2)	6.4(6.1–6.7)	< 0.001
Self-liberation	5.6(2.1)	6.4(1.9)	6.4(6-6.7)		5.6(2.5)	5.7(2.5)	5.7(5.4–5.9)	0.001
Behavioral Processes of change	23.9(5.9)	25(5.5)	25(24.3–25.8)		23.9(7.6)	24(5.7)	24(23.4–24.6)	0.030
Counter conditioning	4.4(2)	5(1.6)	5.1(4.8–5.5)		4.7(2.3)	4.7(2.2)	4.7(4.3–4.8)	0.010
Reinforcement management	4.6(2.2)	4.5(2)	4.2(4-4.5)		4(2)	4(2.1)	4.2(4-4.4)	0.856
Stimulus control	4.1(1.5)	4.4(0.9)	3.3(4.1–4.6)		4(1.5)	4(1)	4(3.8-4)	0.039
Helping relationship	5.1(2.06)	4.8(1.8)	5(4.7–5.3)		5.5(2)	4.8(1.8)	5.5(5.3–5.7)	0.005
Social liberation	6.7(2)	6.9(1.8)	6.9(6.6–7.2)		6.7(2)	6.5(1.9)	5.5(6.3–6.7)	0.054
Decisional balance	4.4(11.9)	10.8(9.7)	11.2(10.3–12.1)		5.1(11)	5.7(10.6)	5.5(4.8–6.9)	0.001
Self efficacy	24(6.4)	28.5(5.5)	28.6(27.9–29.2)		24.1(6.2)	24.5(6.2)	24.5(24-24.9)	< 0.001
Knowledge	17.79(2.19)	22.35(1.95)	22.28(21.79–22.77)		17.59(2.36)	18.35(2.24)	18.30(18.03–18.76)	< 0.001

The values are mean (SD), otherwise described.

SD indicated standard deviation; CI, confidence interval.

*Post intervention mean, adjusted to the baseline measures and related 95% confidence interval was reported.

**P-value was reported from the analysis of covariance to compare groups post intervention, controlling for the baseline values.

Given the adjustment made for the baseline measures of constructs, one month after the educational intervention the mean scores of all the TTM constructs, including cognitive change processes (P < 0.001), behavioral change processes (P = 0.030), decisional balance (P < 0.001) and self-efficacy (P < 0.001), were significantly different between the intervention group and the control group. Similarly, all the subscales (P < 0.05) (except for social liberation and reinforcement management subscale from cognitive and behavioral change processes, respectively) and the variable of knowledge were also significantly different between the intervention group and the control group (Table 5). After the intervention, the mean scores of all constructs and subscales were higher for the intervention group than the control group, except for the helping relationships subscale (Table 5).

## Discussion

The results of the distribution of the stages of change in dairy consumption showed that before the intervention, 86% of the students in the intervention group were inactive and 85% of the students in the control group were inactive. While 63% and 84% of the students in the intervention and control groups were inactive after the intervention, respectively, the statistical test demonstrated a significant difference in the progress of change stages after the intervention between the two groups. The obtained results illustrated a positive and significant progress of the intervention group from the pre-action stages to the action stages after the educational intervention based on the TTM. The results of the current study are consistent with similar studies on the promotion of nutritional behaviors, such as the studies carried out by Rouhani-Tonekaboni et al. [16], and Menezes et al. [23]. In the above mentioned studies, the educational program was shown to be effective in the progress of the change stages. After the educational intervention, more individuals were significantly transferred to higher change stages (preparation, action, maintenance) from the intervention group compared to the control group (P < 0.05).

According to the outcomes of the present study, the mean score of cognitive and behavioral processes was significantly different between the intervention and control groups after the intervention, while it was higher for the intervention group. This is in accordance with the findings offered by Di-Noia et al. [24] and Farhadi and Farhadi [25]. Furthermore, the findings demonstrated that the mean score of cognitive processes, including consciousness raising, dramatic relief, self-re-evaluation, environmental re-evaluation, was significantly different between the intervention and control groups after the intervention, while it was higher for the intervention group.

However, a significant increase was observed in the intervention group compared to the control group in terms of the behavioral processes related to the process of self-liberation and counter conditioning. One of the reasons for this finding is perhaps due to the fact that most of the participants in the present study were in the pre-action stages of behavior change. According to the hypotheses of the TTM, this group used the cognitive processes of behavior change to move and advance in the change stages. In reviewing the literature, interestingly, no similar study was found to assess and examine each subscale of cognitive and behavioral change processes.

As change processes help people progress through the change stages, individuals seek information and explore the benefits of changing and adopting new behaviors in the early stages of behavior change. Therefore, they use cognitive processes more often. In contrast, the individuals entering the active stages of behavior change (action and maintenance) replace unhealthy behaviors with healthy behaviors and attempt to prevent returning to unhealthy behaviors and maintain their new behaviors by applying behavioral processes [12].

In this study, the mean score of the decisional balance construct in dairy consumption was higher for the intervention group, while it was significantly different between both the intervention and control groups after the educational intervention. This construct is the result of two subscales of the perceived pros and cons. Educational intervention was effective in the students’ decisional balance in dairy consumption; in fact, this finding is consistent with Di-Noia’s study [24].

In Hazavehei et al.‘s study, the mean score of benefits in the intervention group was significantly higher than the one in the control group [26]. Also, in a study conducted by Purnarani et al., an educational intervention was an effective factor in decisional balance, and there was a statistically significant difference found between the intervention and control groups after the intervention [27]. According to the TTM, behavior change occurs when the benefits of behavior change are considered more important than its disadvantages. These constructs are especially important in the early stages of behavior change, that is, in the pre-contemplation and contemplation change stages [12]. These results showed that the intervention on the decisional balance construct would contribute to the development of the individuals who were in the early stages of change and would guide them to the higher stages of behavior change. The outcomes of this study also demonstrated a statistically significant difference in the mean score of self-efficacy construct between the intervention and control groups after the intervention. This finding is in accordance with the studies conducted in Iran [27] or in other countries [28–30]. In this regard, self-efficacy was the strongest construct in predicting behavior change and its changes would occur after active participation. The greatest change has often seen in individuals with high levels of self-efficacy in the literature [31, 32]. According to the findings, the individuals in the intervention group made significant and positive progress in dairy consumption stages of change, which would lead to the improvement of self-efficacy.

This research, like all studies, was not without limitations. One of the limitations was related to the concurrence of the study with COVID-19 pandemic, which caused the follow-up period to be considered one month after the end of the educational intervention; therefore, future research can extend the follow-up period to better evaluate the educational outcomes. Another limitation concerned with the duration of each training session due to the pandemic. Finally, the last limitation faced in this study was related to behavior evaluation which was based on the students’ self-report information, because subjective assessments might bring about some bias in data presentation.

Thus, it is suggested that future studies use a combination of self-report methods, direct observation of behavior and parental reports. As designing an educational intervention and presenting a program by educators can be costly and time-consuming, it is suggested that a combination of electronic and printed tutorials with group training based on behavior change models be used. It is suggested that school authorities plan educational interventions to promote dairy consumption under the supervision of health education and health promotion specialists. Educating individuals on the benefits of dairy consumption can reduce some possible barriers as well. Furthermore, promoting self-efficacy using small group educational methods and social media posters on the importance of dairy consumption in school can also be very helpful. The school authorities can also work together to promote the healthy nutritional environment of their schools to support the health of the students. For example, they can improve the distribution of healthy foods such as offering their students packaged milk and they can also reduce the unhealthy quality of the foods offered and served at their school buffets. Finally, the online design of group trainings can reduce the time and space limitations of face-to-face training.

## Conclusions

According to the findings of this study, it seems that appropriate educational programming and implementation based on the TTM can play a significant role in adopting nutritional behaviors of dairy consumption and can change the behavior of dairy consumption in female Iranian students. Given the importance of students’ health in each society and the low cost and effectiveness of the TTM-based nutrition education, it is suggested that more research be designed and implemented based on this model to improve some other nutritional behaviors in students.

## Electronic supplementary material

Below is the link to the electronic supplementary material.

Supplementary Material 1

## Data Availability

The datasets are available from the corresponding author on reasonable request.

## List of abbreviations

WHO: World Health Organization
TTM: Transtheoretical model
CVR: Content validity ratio
CVI: Content validity index
ICC: Intra-class correlation coefficients
ANCOVA: Analysis of covariance
