# Supplementary material for: Promoting dairy consumption behavior in the school setting: an experiment based on the transtheoretical model
Source: BMC Nutr. 2023 Jun 28;9:77. doi: 10.1186/s40795-023-00736-7 (PMC10303894; doi:10.1186/s40795-023-00736-7)
Supplement: Supplementary file 1 — Supplementary Material 1 [file 40795_2023_736_MOESM1_ESM.docx]

**In the name of God**


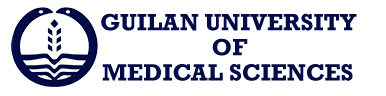


**School of Health**

Hello dear Student

This Questionnaire is designed to conduct research to determine the effect of the educational intervention based on the Trans-theoretical model on the dairy consumption of female students of the 10^th^ and 11^th^ grades of public schools in Someesara city.

If you are willing, please help the researchers in obtaining real information by paying full attention to the questions in the questionnaire and giving honest answers.

Thank you in advance for your sincere cooperation.

**Department of health education and promotion**

**First section**

1. What grade are you in?

a. The tenth□ b.Eleventh**□**

2. How many people are in your family?

a. One b. Two c. Three d. Four and more

3. Where do you live?

a. Village b. city

4.What is your birth rank in the family?

a. First b. second c.Third d. Fourth and more

5. What is your father’s job?

a.Manual worker b. Employee c. Self-employed d. Farmer

6. What is your mather’s job?

a. Housewife b.Manual worker c. Employee d. Farmer e. other

7.What is your father's education level?

a. Illiterate b. Elementary c. Guidance d. High school/diploma e. Upper diploma f. Bachelor's degree g. Master’s degree and higher

8. What is your Mather’s education level?

a. Illiterate b. Elementary c. Guidance d. High school/diploma e. Upper diploma f. Bachelor's degree g. Master’s degree and higher

**Second section**

Dear student, the following questions are about measuring your knowledge about the daily consumption of dairy products. Please read the questions carefully and answer them honestly.

| items | Yes | No | I do not know |
| --- | --- | --- | --- |
| 9. Dairy products include milk, cocoa milk, chocolate milk, buttermilk, yogurt, buttermilk, cheese, cream, ice cream and curd. |  |  |  |
| 10. Dairy products are a good source of calcium needed by the body. |  |  |  |
| 11. Consumption of dairy products strengthens teeth. |  |  |  |
| 12. Consumption of dairy products helps to strengthen bones. |  |  |  |
| 13. If a person does not want to consume milk, she can consume yogurt, buttermilk or cheese instead. |  |  |  |
| 14. Dairy products should be used daily for proper height growth |  |  |  |
| 15. Consuming a sufficient amount of dairy products during adolescence can prevent osteoporosis in the years to come. |  |  |  |
| 16. Cheese is a rich source of calcium. |  |  |  |
| 17. The daily intake of milk for a teenager is two glasses or more to receive calcium. |  |  |  |
| 18. Full-fat milk and yogurt have a higher calcium content than low-fat milk or yogurt. |  |  |  |
| 19. Yogurt is a rich source of calcium. |  |  |  |
| 20. Dairy consumption can help with fitness. |  |  |  |

**Third part**

21. Please read the instructions below carefully and then choose whichever statement is correct about you.

Note: Dairy consumption includes: daily consumption of milk, yogurt, cheese, buttermilk, ice cream and curd. The recommended amount is two to three units per day.

For example each unit of milk is equal to a glass of milk, each unit of yogurt is equal to a bowl of yogurt, each unit of buttermilk is equal to two glasses of buttermilk, each unit of cheese is equal to a match box of cheese matches.

a. I currently consume less than two units of dairy products and I do not plan to increase it in the next six months.

b. I currently consume less than two units of dairy, but I plan to increase it in the next six months.

c. I currently consume less than two units of dairy but plan to increase my intake two to three units per day in the next thirty days.

d. I have been consuming two to three units of dairy products daily for six months.

e. I have been consuming two to three units of dairy products daily for more than six months.

**Fourth section**

The following experiences can affect people's diet habits. Think about the similar experiences you have had in the past month. How many of the following situations have really happened to you? Please, mark the answer that is correct for you.

| Always | Most of the times | Sometimes | Rarely | Never | Over the past month: |
| --- | --- | --- | --- | --- | --- |
|  |  |  |  |  | 1. I was reading about the consumption of dairy products to learn to consume more dairy products. |
|  |  |  |  |  | 2. I paid more attention to the education that was provided about the consumption of dairy products through school, radio and television, etc. |
|  |  |  |  |  | 3. I was afraid of the harm of not consuming dairy products for my health. |
|  |  |  |  |  | 4. I was saddened to hear the statistics of diseases that have occurred in people due to not consuming dairy products. |
|  |  |  |  |  | 5. I knew that if I don't eat enough dairy products, I will make myself sick and others suffer. |
|  |  |  |  |  | 6. I knew that regular consumption of dairy products plays a role in reducing healthcare costs. |
|  |  |  |  |  | 7. I felt better when I regularly consumed dairy products. |
|  |  |  |  |  | 8. I believe that I cannot be a healthy person if I do not eat enough dairy products. |
|  |  |  |  |  | 9.I noticed that many people include dairy products in their diet. |
|  |  |  |  |  | 10. Most restaurants have made it easier for people to consume dairy products outside the home by having dairy products such as buttermilkand yogurt. (buttermilk is a kind of beverage local product) |
|  |  |  |  |  | 11. Instead of consuming chips, puffs, soft drinks and industrial juices, I was choosing dairy products. |
|  |  |  |  |  | 12. Instead of eating unhealthy snacks to satisfy my hunger, or while watching TV, I consumed dairy products. |
|  |  |  |  |  | 13. I encouraged myself when I regularly consumed dairy products daily. |
|  |  |  |  |  | 14. I expected others to encourage me when I consumed more dairy products. |
|  |  |  |  |  | 15. I put posters and pictures of different dairy products in my house or room to remind me to consume them. |
|  |  |  |  |  | 16. I made sure I always had some dairy available. |
|  |  |  |  |  | 17. I had people in my family who encouraged me to consume dairy products. |
|  |  |  |  |  | 18. I tried to eat with friends who had more dairy in their meals. |
|  |  |  |  |  | 19. I told myself that if I try hard enough, I can consume dairy regularly. |
|  |  |  |  |  | 20. I promised myself that by regularly consuming dairy products, I would reduce my health-threatening factors. |

**Fifth section**

This section considers the positive and negative aspects of dairy consumption. Read the following sentences carefully and determine how important each of them is to you when deciding whether or not to consume dairy products.

| item | Not at all important | Slightly important | Somewhat important | Much important | Very much important |
| --- | --- | --- | --- | --- | --- |
| 42. Increasing my daily intake of dairy products helps me have stronger bones. |  |  |  |  |  |
| 43. Consuming more dairy products a day helps reduce the risk of breaking my bones. |  |  |  |  |  |
| 44.By increasing my dairy intake, I have done something for my future health |  |  |  |  |  |
| 45.I have taken care my health by consuming more dairy products daily. |  |  |  |  |  |
| 46. If dairy is available at home, I will consume it. |  |  |  |  |  |
| 47. If I eat enough dairy, my family is less likely to have to worry about a broken bone later. |  |  |  |  |  |
| 48. By consuming more dairy products, I become a good role model for others. |  |  |  |  |  |
| 49. By consuming dairy products, I feel that I have consumed valuable food. |  |  |  |  |  |
| 50. Eating dairy helps control my weight. |  |  |  |  |  |
| 51. Dairy consumption helps to increase my height growth. |  |  |  |  |  |
| 52. Consuming more full-fat dairy products leads to higher blood lipids than when I don't. |  |  |  |  |  |
| 53. Eating more dairy will make me feel fat. |  |  |  |  |  |
| 54. Consuming dairy products causes an increase in calories compared to when I don't consume them. |  |  |  |  |  |
| 55. Eating dairy makes my joints hurt more. |  |  |  |  |  |
| 56. Consuming more dairy products can be harmful to my health. |  |  |  |  |  |
| 57. Consumption of dairy products increases the cost of living. |  |  |  |  |  |
| 58. Consumption of dairy products causes bloating and abdominal pain. |  |  |  |  |  |
| 59. I feel nauseous after drinking milk. |  |  |  |  |  |
| 60. Dairy has an unpleasant taste. |  |  |  |  |  |
| 61. Consumption of dairy products causes trouble for my family. |  |  |  |  |  |
| 62. Dairy products are food for children and sick people. |  |  |  |  |  |
| 63. After consuming dairy products, I feel sleepy and cannot do my activities and homework. |  |  |  |  |  |

**Sixth section**

This section determines your seriousness and perseverance in consuming dairy products.

That is, how confident are you that you can overcome obstacles to consuming dairy products?

Please read the items carefully and mark the answer that is correct for you.

| item | I am not sure at all | I am somewhat surec | I am very sure | I am absolutely sure |
| --- | --- | --- | --- | --- |
| 64. I can start consuming dairy products. |  |  |  |  |
| 65. I can increase my dairy intake. |  |  |  |  |
| 66. I can consume the recommended amount of dairy products. |  |  |  |  |
| 67. I can remind myself to consume dairy. |  |  |  |  |
| 68. I can consume dairy in my dinner. |  |  |  |  |
| 69. I can consume dairy in my snack. |  |  |  |  |
| 70. I can consume dairy with my lunch. |  |  |  |  |
| 71. I can consume dairy products when I eat out with friends. |  |  |  |  |
| 72. I can consume foods containing dairy products (such as porridge, barley soup, etc.). |  |  |  |  |
| 73. I can consume dairy products such as milk, cheese, etc. in my breakfast. |  |  |  |  |

I have completed this questionnaire knowingly and with personal consent.

Signature..................................

Good luck
